# Correlation between Subtypes of *Cryptosporidium parvum* in Humans and Risk

**DOI:** 10.3201/eid1301.060481

**Published:** 2007-01

**Authors:** Paul R. Hunter, Stephen J. Hadfield, Dawn Wilkinson, Iain R. Lake, Florence C.D. Harrison, Rachel M. Chalmers

**Affiliations:** * University of East Anglia, Norwich, United Kingdom; † National Public Health Service for Wales, Swansea, United Kingdom

**Keywords:** Cryptosporidium, microsatellite typing, discriminatory power, zoonoses, research

## Abstract

The 2 main species of *Cryptosporidium* that infect humans are *Cryptosporidium hominis* and *C. parvum*. Here, multilocus fragment analysis of 3 microsatellite loci (ML1, ML2, and gp60) was used to subtype strains from sporadic cases of cryptosporidiosis in Wales and northwest England. Of 72 strains of *C. parvum*, 63 were typeable at all 3 loci, forming 31 subtypes. These strains formed 3 broad clusters, representing 74.6%, 20.6%, and 4.8% of typeable strains. Of 118 *C. hominis* strains, 106 were typeable at all 3 loci, forming 9 subtypes; however, 90% belonged to the same subtype. Analysis with epidemiologic data found an association between strains from case-patients who reported contact with farm animals and individual *C. parvum* microsatellite alleles. The strongest association was with ML1; all strains from case-patients that reported farm animal contact had the same allele (ML1–242). Microsatellite typing of *C. parvum* provides valuable additional information on the epidemiology of this pathogen.

## Abstract 2

Multilocus microsatellite analysis discriminates among *C. parvum* strains and is a potential tool for identifying anthroponotic or zoonotic transmission.

*Cryptosporidium* species are intestinal parasites that infect a variety of animals; *Cryptosporidium hominis* (synonym: *Cryptosporidium parvum* genotype 1) and *C. parvum* (synonym: *C. parvum* genotype 2) are the 2 most commonly identified species that cause disease (cryptosporidiosis) in humans (*1*,*2*). The main symptom of cryptosporidiosis is diarrhea, which may be accompanied by dehydration, weight loss, abdominal pain, fever, nausea, and vomiting (*3*). In England and Wales, ≈5,000 cases are reported annually (*4*). Disease, although lasting for up to 2 weeks, is usually self-limiting in immunocompetent persons but may be chronic and more severe in immunocompromised patients (*5*). Furthermore, *C. hominis* is associated with increased risk of postinfection symptoms (*6*).

*C. hominis* primarily infects humans but has recently been reported to infect a dugong and a lamb, and other animals have been infected experimentally (*7*). By contrast, *C. parvum* naturally infects several animal species that serve as reservoirs for zoonotic infection, including cattle, sheep, goats, and deer (*7*).

Several methods have been described by different research groups to investigate intraspecies variation within the genus *Cryptosporidium,* including microsatellite sequence analysis (*8*–*11*), minisatellite and microsatellite PCR fragment length analysis (*12*,*13*), single-strand conformation polymorphism analysis (*14*), gp60 sequence analysis (*15*,*16*), and telomere sequence analysis (*17*,*18*). A recent study that used minisatellite and microsatellite fragment analysis identified some *C. parvum* clones that may not be zoonotic (*12*,*13*); this study compared isolates from humans and bovines in a single Scottish county. However, no epidemiologic data were presented on case-patients. In the study described here, we investigated the subtypes of *C. parvum* and *C. hominis* and tested the association of subtypes with known epidemiologic factors.

## Materials and Methods

### Strains

The strains included in this analysis were collected during the case-control study of human cryptosporidiosis in Wales and northwest England (*19*). This study is to date the only case control-study of risk factors for cryptosporidiosis with species identification of infecting strains. Some 427 case-patients and controls were surveyed by mail questionnaire. The key findings were that travel abroad and changing diapers of children <5 years of age were associated with risk for *C. hominis* infections. For *C. parvum*, touching farm animals was associated with illness but eating raw vegetables and tomatoes was strongly negatively associated with illness.

As part of that study, clinical laboratories were encouraged to send fecal samples positive for *Cryptosporidium* by microscopy to the UK Cryptosporidium Reference Unit in Swansea. Confirmation that samples were positive by microscopy was performed when required by using a modified Ziehl-Neelsen method as described by Casemore et al. (*20*). To extract *Cryptosporidium* DNA from microscopy-positive feces, oocysts were first separated from fecal matter by saturated-salt-solution centrifugation as described by Elwin et al. (*21*). The oocyst suspension was then incubated at 100°C for 60 min, digested with proteinase K and lysis buffer, and purified by using QIAamp DNA Mini Kit spin columns (QIAGEN Ltd, Crawly, UK) as described previously (*2*). DNA was stored at –20°C before species determination and subtyping, when appropriate.

### Identification of Species or Genotype by PCR–Restriction Fragment Length Polymorphism Analysis (PCR-RFLP)

*Cryptosporidium* sp. was determined by PCR-RFLP analysis of the *Cryptosporidium* oocyst wall protein (COWP) and small subunit (SSU) rRNA genes using methods based on those described by Spano et al. (*22*) and Xiao et al. (*23*), respectively. For PCR-RFLP analysis of the COWP gene, PCR was carried out by using the forward primer 5′-GTAGATAATGGAAGAGATTGTG-3′ and reverse primer 5′-GGACTGAAATACAGGCATTATCTTG-3′ to produce an amplicon of ≈550 bp. The PCR products were digested by using the restriction enzyme *Rsa*I to differentiate between most *Cryptosporidium* spp.

For nested PCR-RFLP analysis of the SSU rRNA gene, the primary PCR produced fragments of ≈1,325 bp by using the forward primer 5′-TTCTAGAGCTA ATACATGCG-3′ and the reverse primer 5′-CCCATTTCCTTCGAAACAGGA-3′. The secondary PCR, which produced fragments of ≈830 bp, used the forward primer 5′-GGAAGGGTTGTATTTATTAGATAAAG-3′ and the reverse primer 5′-AAGG AGTAAGGAACAACCTCCA-3′. The products of the secondary PCR were digested with *Ssp*I and *Vsp*I. Digested fragments from SSU rRNA and COWP genes were separated by electrophoresis on 3% agarose gels, visualized by SYBR Green I (Sigma, Gillingham, UK) staining, and images were recorded with a digital imaging system (Alpha Imager, Kodak, Hemel Hempstead, UK).

### Confirmation of Species or Genotype by SSU rRNA Gene Sequence Analysis

After PCR-RFLP analysis, unusual species and equivocal samples were confirmed by amplifying a fragment of the SSU rRNA gene and DNA sequencing in both directions. Briefly, amplicons of ≈830 bp were produced from each sample by using the nested primer set described above (*23*), and an ≈298-bp fragment was sequenced (Genetic Research Instrumentation, Braintree, UK) by using the forward primer 5′-AGTGACAAGAAATAACA ATACAGG-3′ and the reverse primer 5′-CCTGCTTTAAGCACTCTAATTTTC-3′ (*24*). The forward and reverse sequences of these fragments were then aligned and analyzed with a CEQ 8000 Genetic Analysis System (Beckman Coulter, High Wycombe, UK) to obtain a consensus sequence. This sequence was then compared with all GenBank, EMBL, DDBJ, and PDB sequences by using the National Center for Biotechnology Information BLASTN tool (available from http://www.ncbi.nlm.nih.gov/BLAST/).

### Analysis of *C. hominis* and *C. parvum* Subtypes

Subtypes were identified by using a multilocus fragment-size–analysis approach to target 3 microsatellite markers (ML1, ML2, and gp60 [synonymous with gp15]) as previously described (*25*). The ML1 fragment was amplified by using the forward primer 5′-CTAAAAATGGTGGAGAATATTC-3′ and the reverse primer 5′-CAACA AAATCTATATCCTC-3′ (*9*,*10*). The ML2 fragment was amplified by using the forward primer 5′-CAATGTAAGTTTACTTATGATTAT-3′ and the reverse primer 5′-CGACTATAAAGATGAGAGAAG-3′ (*10*). The gp60 fragment was amplified by using the forward primer 5′-GCCGTTCCACTCAGAGGAAC-3′ and the reverse primer 5′-CCACATTACAAATGAAGTGCCGC-3′ (*12*). Reverse primers were supplied that were labeled with Beckman Coulter WellRED D3 dye (Proligo**,** Paris, France). The 50-μL PCR mixture for each primer set contained PCR buffer (QIAGEN Ltd), 2.5 mmol/L of MgCl_2_, 200 μmol/L of each dNTP, 500 nmol/L of each primer, 2.5 U of HotStar *Taq* DNA polymerase (QIAGEN Ltd), and 5 μL of template DNA. The cycling conditions for each PCR were an initial denaturing step of 15 min at 95°C, then 40 cycles of 95°C for 50 s, 50°C (60°C for gp60) for 50 s, and 72°C for 60 s before a final extension of 10 min at 72°C. The fragment sizes of amplified products were then analyzed with a CEQ 8000 Genetic Analysis System (Beckman Coulter). Allele nomenclature was based on the median fragment size of each natural group rounded to the nearest probable base pair number. The combined results of fragment-size analysis at all 3 markers were used to create a multilocus fragment type for subtypes within *C. parvum* and *C. hominis* as described elsewhere (*25*,*26*).

### Statistical Analysis

Data analysis was carried out by using SPSS 12.0 (SPSS Inc., Chicago, IL, USA). Subclusters were identified by using the SPSS clustering algorithm, a hierarchical algorithm that clusters strains and other clusters together on the basis of their similarity.

χ^2^ tests (or Fisher exact test when data were sparse) were used to identify significant trends between *C. parvum* cluster 1 and *C. parvum* clusters 2 and 3 combined, with epidemiologic parameters. A final multivariable model was derived by using logistic regression as previously described (*19*) and including all the different strains of *C. parvum*; the model was recalculated including only the strains that possessed the ML1–242 allele.

The Hunter-Gaston index of discriminatory power was calculated by using StatsDirect (*27*). This index was proposed as a measure of the discriminatory power of microbial typing schemes. By using the typing scheme under investigation, it calculates the probability of randomly picking 2 unrelated strains and finding them to be different.

## Results

A total of 190 sporadic strains of *Cryptosporidium* were included in this analysis: 118 were *C. hominis,* of which 106 were typeable at all 3 microsatellite loci; 72 were *C. parvum*, of which 63 were typeable at all 3 loci. The distribution of these types is shown in Table 1.

**Table 1:** Distribution of multilocus fragment types **(**MLFTs) for *Cryptosporidim* strains typeable at all 3 loci

Species/MLFT	No. strains	%	ML1 allele	ML2 allele	gp60 allele
*C. hominis*					
H1	95	89.6	233	180	371
H2	3	2.8	239	180	371
H3	2	1.9	242	180	371
H4	1	0.9	224	180	371
H5	1	0.9	233	180	407
H6	1	0.9	233	180	353
H7	1	0.9	218	180	371
H8	1	0.9	218	180	413
H9	1	0.9	233	180	341
*C. parvum*					
P1	8	12.7	242	229	341
P2	5	7.9	242	229	338
P3	2	3.2	227	193	329
P4	2	3.2	227	195	338
P5	6	9.5	242	231	341
P6	4	6.3	242	233	338
P7	6	9.5	242	231	338
P8	3	4.8	242	233	341
P9	1	1.6	242	225	341
P10	1	1.6	242	227	338
P11	1	1.6	242	229	332
P12	1	1.6	242	229	359
P13	1	1.6	242	229	347
P14	1	1.6	242	231	356
P16	1	1.6	242	231	344
P17	3	4.8	242	231	347
P18	1	1.6	242	233	347
P19	1	1.6	242	235	338
P20	1	1.6	242	237	341
P21	1	1.6	242	231	350
P22	1	1.6	227	193	320
P23	2	3.2	227	195	326
P24	1	1.6	227	223	332
P25	2	3.2	227	197	311
P26	1	1.6	227	231	341
P27	1	1.6	227	195	353
P28	1	1.6	227	193	326
P29	1	1.6	227	193	329
P30	1	1.6	227	195	332
P31	1	1.6	227	229	326
P32	1	1.6	242	237	338

Of the 106 strains of *C. hominis* typeable at all 3 loci, 95 (90%) were indistinguishable at all 3 loci, having the ML1 allele 233 (ML1–233), ML2–180, and gp60–371. This lack of diversity of *C. hominis* as demonstrated by these 3 markers did not allow further analysis.

Much greater diversity in allele size at all 3 microsatellite loci was displayed by *C. parvum* than by *C. hominis*. The discriminatory power of the 3-loci typing method for *C. parvum* using the Hunter-Gaston index of discriminatory power was 0.957 (95% confidence interval [CI] 0.937–0.977). For *C. hominis,* the discriminatory power was 0.197 (95% CI 0.096–0.298).

The Appendix Figure shows a 3-dimensional scatterplot of the strains of *C. parvum*. Considerable variation can be seen in microsatellite length, and 3 broad subclusters are identifiable. Strains belonging to the 2 smaller clusters had the same ML1–227 allele, whereas all strains belonging to the larger cluster had the ML1–242 allele.

**Figure 1:**
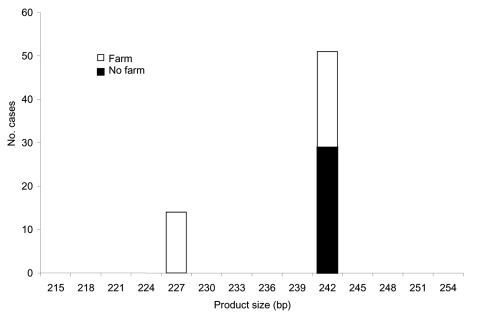
Product size at microsatellite locus ML1 with number of *Cryptosporidium parvum* case-patients who touched or handled farm animals before onset of illness.

**Figure 2:**
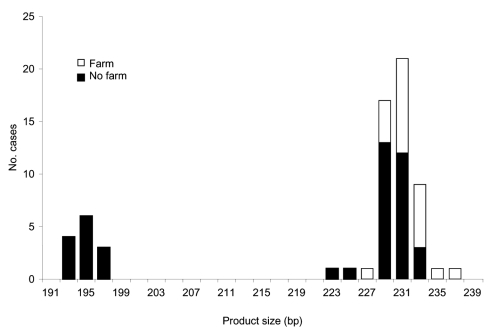
Product size at microsatellite locus ML2 with number of *Cryptosporidium parvum* case-patients who touched or handled farm animals before onset of illness.

**Figure 3:**
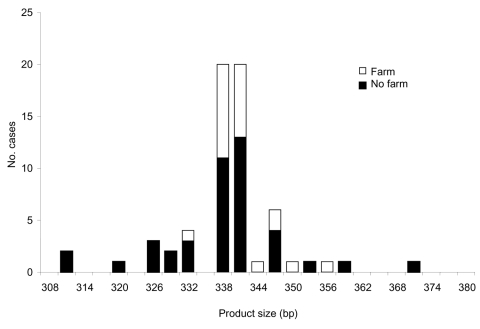
Product size at microsatellite locus gp60 with number of *Cryptosporidium parvum* case-patients who touched or handled farm animals before onset of illness.

We further looked at the association between polymorphisms at the 3 loci and reported case-patient contact with animals. For this analysis, all strains were included, whether or not they were typeable at all 3 loci. Significantly more persons with strains with ML1–242 (22/52, 43%) had touched or handled farm animals than those with ML1–227 strains (0/14, 0%) (Mann-Whitney U test, p = 0.000 (Figure 1). Similarly, at ML2, significantly more strains with alleles between 223 and 237 (42%, 22/52) were from case-patients who had touched or handled farm animals than were strains with alleles 193 and 197 (0%, 0/13) (Mann-Whitney U test, p = 0.000) (Figure 2). Alleles of gp60 (Figure 3) varied from 311 to 371 bp and peaked at 340 to 341 bp. Case-patients who had contact with farm animals yielded significantly greater product sizes at this locus than those who reported no animal contact before onset of illness (Mann-Whitney U test, p = 0.003).

To test further the association between the ML1–242 polymorphism and contact with animals, the final logistic regression model for *C. parvum* presented in our earlier article (*19*) was re-run but included only those strains with the 242-bp allele. The positive association with farm animals and the negative associations with eating raw vegetables all are stronger in the model with just ML1–242 allele strains than in the model containing all *C. parvum* strains (Table 2).

**Table 2:** Logistic regression model from case-control study (*19*) showing final model from original study and recalculated using only those strains with the ML1–242 polymorphism as cases*

Cases/variable	Cases, n (%)	Controls, n (%)	Odds ratio	95% CI	p value
All *Cryptosporidium parvum* strains					
Touch or handle any farm animals					
Yes	24 (34)	43 (11)	2.653	1.113–6.323	0.028
No	47	348			
Eat tomatoes					
Yes	24 (36)	249 (50)	0.317	0.140–0.719	0.005
No	43	246			
Eat raw vegetables					
Yes	7 (12)	157 (44)	0.222	0.086–0.572	0.001
No	51	196			
Only ML1–242 strains					
Touch or handle any farm animals					
Yes	21 (43)	43 (11)			
No	28	348	3.810	1.444–10.049	0.007
Eat tomatoes					
Yes	17 (37)	249 (50)	0.425	0.164–1.104	0.079
No	29	246			
Eat raw vegetables					
Yes	4 (10)	157 (44)	0.141	0.042–0.474	0.001
No	37	196			

*CI, confidence interval. Also included in the models were age and Health Authority of residence.

Each typeable strain was also categorized by local environment, based on postal code of patient’s residence. These categories were urban, town or town fringe, village, and hamlet or isolated dwelling. The attack rates per 100,000 population for each of the 2 ML1 types of *C. parvum* are shown in Table 3. The incidence of ML1–242 strains increased as the home environment became increasingly rural, whereas ML1–227 strains were largely restricted to urban and town environments (Mann-Whitney U test, p = 0.005).

**Table 3:** Association between subtype number and attack rate per 100,000 population and residential land use

Residential land use	ML1–242		ML1–227	
	No.	Attack rate	No.	Attack rate
Urban	16	0.21	10	0.13
Town and fringe	10	1.31	4	0.52
Village	14	2.72	0	0.00
Hamlet and isolated dwellings	12	3.60	1	0.30

## Discussion

At these 3 microsatellite loci, much greater genetic diversity was detected among *C. parvum* strains than among *C. hominis* strains. For *C. parvum* the 3 loci were highly discriminatory (Hunter-Gaston index 0.957), but for *C. hominis,* they were poorly discriminatory (0.197). These 3 loci by themselves are unlikely to be sufficient for subtyping *C. hominis* but are adequate for subtyping *C. parvum*.

Using all 3 loci, the typeability for *C. hominis* was 90% and for *C. parvum* 87.5%. The presence of nontypeable strains in any one of the 3 single loci reduced the overall typeability and therefore discriminatory power of the typing method. However, strains that did not type at every locus could still be compared. For example, 70 (96%) strains of *C. parvum* were typed at the ML1 locus, which improved the power of analyses using just this locus. We are unable to say whether nontyping at a particular locus was because of an unusual allele or because of the sensitivity of the method.

The low diversity of *C. hominis* is to be expected because it is a species-specific parasite. Hunter and Fraser (*28*) noted that species adapted to single host species were likely to be less genetically diverse than those with a wider host range, as predicted by the theory of adaptive polymorphism. Greater genetic variation was also found among *C. parvum* (type 2) than *C. hominis* (type 1) isolates in a previous study that used minisatellite and microsatellite loci (*12*). This apparently low genetic diversity among strains of *C. hominis* might make it difficult to develop discriminatory and reproducible typing methods for *C. hominis.* However, recent investigation of isolates from global sources at multiple minisatellite and microsatellite loci showed increased polymorphism, particularly over many minisatellite loci (*29*). On the other hand, the use of only 3 loci gives good discriminatory power for *C. parvum*.

Using just 3 microsatellite loci, we have shown that 3 major groupings of *C. parvum* can be found, which supports the similar findings of Mallon et al. (*12*), who used 7 loci. These researchers reported that the largest cluster contained strains isolated from both humans and animals, while the 2 smaller clusters contained strains isolated only from humans. In our study, all strains isolated from persons reporting contact with animals came from cluster 1, which supports the suggestion of 2 clones of human-adapted strains of *C. parvum*.

The most intriguing finding was that of an association between strains of *C. parvum* that may be human-adapted or zoonotic and particular alleles of the microsatellites. While this association included all 3 loci, the strongest association was with alleles at the ML1 locus. This observation was even more dramatic, given that only 2 alleles were found at this locus. None of the case-patients whose strains yielded ML1–227 reported contact with farm animals, while 43% of those whose strains yielded ML1–242 reported such contact. This finding is strengthened by the observation that most of the case-patients yielding cluster 2 or 3 strains were more likely to live in urban areas where the possibilities for animal contact are lower than for those yielding cluster 1 strains. In a related study, all 28 strains isolated from animals were ML1–242, which further supports this hypothesis (*26*,*30*).

Although the ML2 locus is more variable than the ML1 locus, the 2 loci correlate very closely. This linkage disequilibrium between the 2 loci has already been noted by other researchers (*10*), although we must emphasize that our results differ from those of Cacciò et al. (*10*), who detected 3 alleles at the ML1 locus (ML1–238, ML1–226, and ML1–220). By sequencing PCR products, these authors also found all 3 alleles in isolates from animals. These discrepancies are not likely to be due to the different methods used for sizing of PCR fragments.

We cannot yet conclude that our findings indicate human-adapted strains of *C. parvum* exist or if all strains are potentially zoonotic. ML1–227 strains do not appear to be zoonotic in the United Kingdom but have been identified as such by other workers in Italy (*10*), for example. If such strains are zoonotic in other countries, they likely would have spread into the UK human population through imported foods or during foreign travel and subsequently spread among humans. However, they may not have yet made the transition to UK animals.

Microsatellite fragment analysis of *C. parvum* would appear to provide a discriminatory and rapid means of distinguishing strains. This technique would be useful in outbreak settings to determine whether outbreaks were due to single or multiple strains and, if the former, may indicate the source of contamination. The microsatellites used in this work would not be discriminatory enough for routine use for *C. hominis,* although others may prove to be of more value.

## Supplementary Material

Appendix Figure Three-dimensional scatter plot of Cryptosporidium parvum strains typeable at all 3 loci.
