# Supplementary figures and images for: Correlation between Subtypes of Cryptosporidium parvum in Humans and Risk
Source: Emerg Infect Dis. 2007 Jan;13(1):82–8. doi: 10.3201/eid1301.060481 (PMC2725800; doi:10.3201/eid1301.060481)

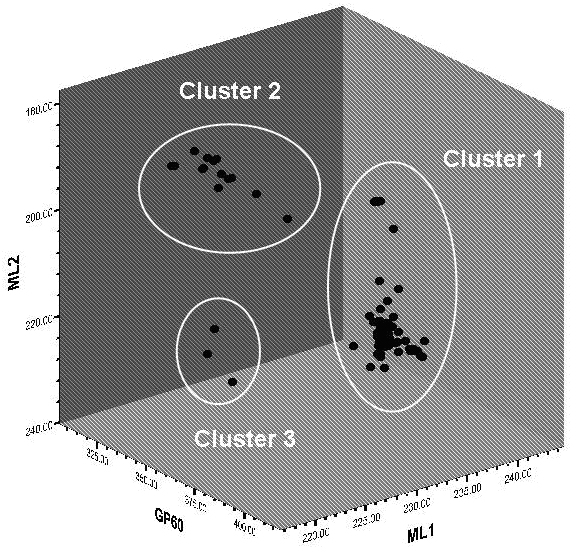

Supplement: Appendix Figure — Three-dimensional scatter plot of Cryptosporidium parvum strains typeable at all 3 loci. [file 06-0481_app-s1.gif]
